# Assessment of the relationship of basal serum anti-mullerian hormone levels with oocyte quality and pregnancy outcomes in patients undergoing ICSI

**Published:** 2015-04

**Authors:** Gültekin Adanaş Aydın, Arzu Yavuz, Hasan Terzi, Tayfun Kutlu

**Affiliations:** 1 *Department of Obstetrics and Gynecology, Bursa Çekirge State Hospital, Bursa, Turkey.*; 2 *Department of Obstetrics and Gynecology, Kocaeli Derince Education and Research Hospital, Kocaeli, Turkey.*; 3 *IVF Clinic, Zeynep Kamil Women's and Children's Hospital, Istanbul, Turkey.*

**Keywords:** *Anti-Mullerian hormone*, *Ovary reserve*, *Oocyte quality*

## Abstract

**Background:**

Anti-Mullerian hormone (AMH) is constantly secreted during menstrual cycles and may offer several advantages over traditional biomarkers of ovarian reserve.

**Objective:**

To assess the relationship of anti-Mullerian hormone (AMH) values, which are used to evaluate ovary reserves, with oocyte and embryo quality and with ART outcomes in patients undergoing intra-cytoplasmic sperm injection (ICSI).

**Materials and Methods:**

This cross sectional study was performed using 50 women undergoing ICSI in IVF center of Zeynep Kamil Women's and Children's Hospital, İstanbul, Turkey. All patients received the long protocol. Follicle-stimulating hormone, luteinizing hormone, estradiol, and AMH levels were measured and antral follicle counts were obtained on the 3^rd^ day of menstruation. A cut-off value based on the number of oocytes was determined for AMH, and women were evaluated after being divided into two groups as bad responders and good responders, according to their AMH levels.

**Results:**

Twelve (27.3%) women were in bad responders group and 32 (72.7%) women were in good responders group. AMH measurements were statistically significantly different between the two groups (p<0.01). Based on this significance, the researchers used ROC analysis to estimate a cut-off point for AMH. The researchers detected the good responders with an AMH level 1.90 or above, with 87.50% sensitivity, 66.67% specificity, 87.50% positive prediction, and 66.67% negative prediction (AUC=0.777, p<0.01).

**Conclusion:**

Basal AMH levels can be used as an indicator to determine the ovarian response in women undergoing ICSI. AMH can be used to predict the number of mature oocytes that can be collected during treatment and the number of oocytes that can be fertilized. However, AMH is not a valuable tool to evaluate oocyte quality, the development of high-quality embryos, or pregnancy conception.

## Introduction

Assisted reproductive technology (ART) is an expensive, time-consuming, and stressful treatment approach for patients. For assisted reproductive technology to be successful patients should be adequately evaluated and the correct ART should be selected. The ovary reserve is associated with oocyte number and quality, and it represents the reproductive potential of the woman. While various methods have been developed to evaluate the ovary reserve, few methods are currently available to assess oocyte quality. Methods such as polar body biopsy and examining the oocyte cumulus are time-consuming and technically difficult; thus, they are not routinely performed (1).

The anti-Mullerian hormone (AMH) is a member of the transforming growth factor β family (2). It takes the form of a dimeric glycoprotein and plays roles in tissue growth and differentiation. AMH is secreted by the granulosa cells in the preantral and antral follicles in the ovaries. AMH secretion decreases as antral follicles start to grow, and it stops when a follicle reaches 8 mm or undergoes atresia (3). AMH plays a role in regulating ovarian activity. Additionally, it inhibits initiation of the development of primordial follicles and the selection of a high number of follicles by decreasing the follicles’ sensitivity to follicle stimulating hormone (FSH) (4).

Unlike estradiol (E_2_), inhibin B, and FSH, AMH is constantly secreted during menstrual cycles. Since AMH levels are associated with the number of antral follicles, they are important in detecting ovarian response during controlled ovarian hyper-stimulation (5, 6). Moreover, ovarian response does not only reflect the number of primordial follicles but also shows the oocyte quality (7). This cross sectional study aims to assess the relationship of AMH levels with oocyte and embryo quality and with ART outcomes.

## Materials and methods

This cross sectional study was performed using 50 women from the IVF/ICSI program at the In-Vitro Fertilization Center of Zeynep Kamil Gynecologic and Pediatric Training and Research Hospital, Istanbul, Turkey; the participants involved women receiving treatment between May 2009 and December 2009. The study protocol was performed according to the Helsinki Committee requirements and was approved by the institutional review board of Zeynep Kamil Women's and Children's Hospital. Written informed consent was obtained from all subjects before entering the study.

Women between the ages of 24 and 39 years were included in this study. Women with basal FSH levels under 15 IU/L, TSH, and prolactin (PRL) levels within normal limits (2-29 ng/ml) and having their first ICSI trial were included. Women with a history of previous ovarian surgeries, polycystic ovarian syndrome and premature ovarian failure were excluded. ICSI was applied to 25 women due to a male factor, 14 women due to unexplained infertility, and 5 women due to tubal and peritoneal reasons. To measure AMH levels, 5 mL of total blood was obtained from the women on the third day of menstruation and centrifuged for 10 minutes at 3500 revolutions. The obtained serum samples were stored at -80^o^C in 1.5 mL polypropylene tubes.

All serum samples that were stored since December 2009 were analyzed using the ELISA method using the ACTIVE MIS/AMH ELISA DSL-10-14400 kit from Diagnostic Systems Laboratories (Texas, USA). The long protocol was applied to suppress the menstrual cycle. Subcutaneous administration of 0.5 mg leuprolide acetate and 0.1 mg triptorelin acetate was initiated on the 21^st^ day of the cycle. Serum E_2_ levels measured below 50 pg/ml and ultrasonography endometrium thicknesses below 5 mm measured on the second day of menstruation were considered down- regulation. Gonadotropin treatment was subsequently initiated.

The treatment was initiated with r-FSH (Gonal-F, Serono, Istanbul, Turkey) or human menopausal gonadotropins (Menogon, Ferring Pharmaceuticals, Istanbul, Turkey) at doses between 150-450 IU/day. After achieving three follicles of 17 mm, patients received intramuscular injections of 10000 IU hCG or subcutaneous injections of 250 mcg recombinant hCG. Follicle aspiration was performed after 35 hours and was guided by ultrasound under anesthesia in sterile conditions. Mature and immature oocytes were determined in the obtained follicle plasma; mature oocytes were separated (G-1TM version 3, Vitrolife, Goteborg, Sweden; G-2 TM version 3, Vitrolife Plus) and transferred to the culture medium. Following ICSI, all the embryos were evaluated based on the Z scoring system.

Embryos with two, three, or four cells having equal blastomeres without fragmentation on the second day and embryos with six, seven, or eight cells having equal blastomeres without fragmentation on the third day were evaluated as Grade 1. Embryos with equal blastomeres but having fragmentation at 20% or below were scored as Grade 2. Embryos that were Grade 1 or Grade 2 and those reaching the blastocyst stage on the 5^th^ day (according to microscopic examination done before transfer) were transferred within 48-120 hr. Serum β-hCG levels were measured 12-14 days after transfer to assess pregnancy conception. Following initiation of the gonadotropin agonist therapy, a >20 mm cyst was detected in one patient during TVUSI performed on 2^nd^ day of the menstruation and treatment was terminated.

Treatment was delayed for one woman who had endometrial polyps and one woman who had an adnexal cyst. In line with the literature, the researchers assessed treatment success based on the number of collected oocytes. Of the women, 32 with five or more oocytes after treatment were evaluated as good responders and 12 women with less than five oocytes were evaluated as bad responders. A cut-off value based on the number of oocytes was determined for AMH, and women were evaluated after being divided into two groups according to their AMH levels.

**Statistical analysis**

To evaluate the findings of this study, NCSS 2007 & PASS 2008 Statistical Software (Utah, USA) were used to conduct statistical analyses. Study data were evaluated based on descriptive statistics (mean, standard deviation) and to assess the quantitative data; parameters showing normal distribution were compared between groups using the Student’s t-test. The Mann-Whitney U-test was used to compare the parameters that did not fit a normal distribution between groups. Qualitative data were compared using the chi-square test or Fisher’s exact chi-square test. ROS analysis and diagnosis screening tests were used to determine the cut-off point. The results were evaluated based on a 95% confidence interval at a significance level of p<0.05.

## Results

The study was performed using 44 cases. Table I shows the distribution of the patients’ clinical characteristics. The authors evaluated the women in two groups based on the number of oocytes collected (<5 and ≥5). Within the group, 12 women (27.3%) had less than five oocytes collected, and 32 patients (72.7%) had more than five oocytes collected. The mean±SD AMH level was 1.87±1.40 in the group with less than five oocytes collected and 3.17±1.90 in the group with more than five oocytes collected. Based on the classification of the number of collected oocytes, AMH levels showed a statistically significant difference (p<0.01). 

AMH levels were significantly higher in the group with five or more collected oocytes. Based on this significance, the authors estimated a cut-off point for AMH. ROC analysis was used to estimate the cut-off point for AMH. The authors detected five or more oocytes in the patients with AMH levels of 1.90 or above, with 87.50% sensitivity, 66.67% specificity, 87.50% positive prediction, and 66.67% negative prediction (AUC=0.777, p<0.01) (Figure 1).

When the AMH’s predictive value for biochemical pregnancy, the number of oocytes collected, and the number of mature oocytes were compared, the highest predictive value was for the number of mature oocytes (Table II). The predictive value for biochemical pregnancy was not significant. When the researchers evaluated the patients based on the estimated AMH cut-off value, the basal FSH values were lower, whereas the antral follicle counts, the number of collected and mature oocytes, and the number of fertilized oocytes were significantly higher in the group with higher AMH values.

The rate of high-quality embryos was not significantly different. Biochemical pregnancy was seen in 25% of the patients in the group with AMH levels below 1.90 ng/mL while this rate was 51.6% in the group with AMH levels 1.90 ng/mL or above. This difference was not statistically significant. When the morphological characteristics of the mature oocytes collected were compared, no significant difference was detected between the two groups except for the wide perivitelline space.

**Table I:** Distribution of clinical characteristics (n=44)

	**Min-Max **	**Median (Mean ± SD)**
Infertility duration (Year)	2-18	8.34 ± 3.77
Basal E_2 _(pg/mL)	20-139	48.32 ± 21.03
Basal FSH (IU/L)	4-11	6.91 ± 2.38
AMH (ng/mL)	0.51-11.62	2.81 ± 1.91
Antral follicle count	4-22	13.32 ± 4.14
E_2 _at day 5	10-2058	425.64 ± 368.67
E_2_ on the day of hCG	491-3958	2196.41 ± 981.12

E_2_: Estradiol

FSH: Follicle-stimulating hormone

AMH; anti-Mullerian hormone

**Table II:** Evaluations based on AMH classification

	**AMH**		**p-value**
	**<1.90** **ng/ml (n=12)**	**≥1.90** **ng/ml (n=32)**	
Age (Year)	33.67 ± 3.93	31.47 ± 3.77	0.09+
Duration of infertility (Year)	8.42 ± 4.16	8.31 ± 3.68	0.77++
Basal FSH (IU/L)	8.34 ± 3.17	6.43 ± 1.77	0.04++
Antral follicle count	10.08 ± 4.14	14.94 ± 4.73	0.002++
Basal E_2_ (pg/ml)	57.19 ± 30.73	45.00 ± 15.38	0.17++
Fertilized oocyte count	1.67 ± 1.72	4.66 ± 2.98	0.002++
High-quality embryos	1.25 ± 1.21	2.03 ± 1.47	0.11++
Number of oocytes collected	3.58 ± 2.42	9.31 ± 4.08	0.001
Number of mature oocytes	2.83 ± 2.12	7.63 ± 3.80	0.001

+ Student’s *t*-test

++ Mann-Whitney U-test

Chi-square test

**Table III:** Distribution of the oocyte characteristics between the two groups

	**AMH**		**p-value**
	**<1.90 ** **ng/ml ** **(n= 12)** **n(%)**	**≥1.90 ** **ng/ml ** **(n= 32)** **n(%)**	
Oocytes collected	43	298	
Mature oocytes	34 (79.0)	244 (81.8)	0.65
Normal oocytes	4 (11.8)	50 (20.5)	0.22
Abnormalities	30 (88.2)	194 (79.5)	
Intense PVD	8 (26.7)	38 (19.6)	0.37
SER	1 (3.3)	1 (0.5)	0.25
IC	7 (23.3)	25 (12.9)	0.12
GPVS	6 (20.0)	13 (6.7)	0.01
PPB	4 (13.3)	27 (13.9)	0.93
Thick zoster	12 (40.0)	94 (48.5)	0.38
Central granulation	11 (36.7)	63 (32.5)	0.65
Small PV	0	5 (2.6)	1.00
Vacuolization	0	2 (1)	1.00
Double Polarity	0	1 (0.5)	1.00

Chi-square test

PVD: Perivitelline debris

PV: Small perivitelline space

SER: SER appearance in the cytoplasm

IC: Inclusion particle

GPVS: Wide perivitelline space

PPB: Fragmented polar body

## Discussion

The ovary reserve shows a woman’s reproductive potential and reflects the primordial follicle count and the oocyte quality. Follicle development in the ovaries as a response to gonadotropins may be defined as the ovary reserve. Various tests have been developed to assess the ovary reserve. Parameters such as serum basal FSH, E_2_, inhibin B, pre-treatment ovary volume, and antral follicle count have been evaluated to that end (8). The value of the anti-Mullerian hormone in determining the ovary reserve has begun to be better understood in recent years (5,9,10).

Ficicioglu *et al* found a relationship between antral follicle counts and the number of oocytes collected in their study. They reported that AMH levels reflected the extent of the antral follicle pool and concluded that the AMH level can be used as a marker to determine the number of oocytes to be collected with controlled ovarian stimulation. In the same study, they found that basal AMH levels, antral follicle counts, and E_2_ levels on the hCG day were lower in the group with less than five oocytes collected (10).

When the researchers in the present study divided the patients into groups based on their AMH levels, they found a relationship between AMH and the number of antral follicles counted during the TVUSI performed on the second or third day of the pre-treatment cycle. The antral follicle counts were significantly higher in the group with higher AMH values. While the basal FSH levels used to evaluate ovary reserves were lower in the group with higher AMH levels, the basal E_2_ levels were similar. Age, another important parameter in evaluating ovary reserves, was not significantly different between the two groups. Seifer *et al* found a relationship between early follicular phase serum AMH levels and the number of oocytes obtained after the induction of ovulation. Specifically, they collected a higher number of oocytes and more mature oocytes in patients with higher serum AMH levels (5).

In study performed by Hazout *et al* AMH demonstrated a better correlation with the number of oocytes collected and antral follicle counts compared to inhibin B and FSH (11). The present study divided patients into two groups according to the number of oocytes collected during treatment. The number of mature oocytes collected during treatment was significantly higher in the group of good responders. AMH, inhibin B, FSH levels, and antral follicle counts are parameters used to predict pregnancy conception. In their study, Van Rooij *et al* did not demonstrate a relationship between FSH, E_2_, and inhibin B levels and pregnancy conception (6).

While some researchers did not find any relationship between basal AMH levels and a pregnancy prediction others showed that higher AMH levels were significantly associated with the increased probability of pregnancy conception (10,12-14). Smeenk *et al* reported a relationship between AMH levels and ovary response, but they did not discover a relationship between AMH levels and embryo quality or pregnancy conception. They demonstrated that the other ovary reserve tests were weakly related to oocyte count, embryo count, embryo quality, and pregnancy conception (13).

While some studies reported a positive correlation between AMH levels and embryo scoring, opposing findings have also been reported (12,15). Takahashi *et al* reported that inhibin B, E_2_, and progesterone levels measured in the follicle plasma are not sensitive markers of embryo quality. They also did not find a statistically significant relationship between AMH levels measured on the oocyte pick-up day and the number of high- quality embryos or the fertilization rate (16). In the present study, the researchers did not find a significant difference in pregnancy rates when the two groups with high or low AMH levels were compared. While the number of fertilized oocytes was higher in the group with high AMH levels, the development of high- quality embryos, seen as an effective factor in pregnancy conception, was similar between the two groups.

The ovary reserve is related to both the quantity and quality of the ovary follicle pool. Although direct measurement of the primordial follicle pool is not possible, the antral follicle count is known to be proportional to the size of the primordial follicle pool. For this reason, the antral follicle count is believed to reflect the quantitative changes that may occur in ovaries with age. Unfortunately, there is no marker currently available to directly assess oocyte quality. Ebner *et al* performed an early study focusing on this area in which they assessed oocyte quality according to AMH levels. AMH is released by granulosa cells during the early follicular phase. Decreased AMH levels may be associated with impaired secretions from granulosa cells and may result in irreversible damage to the germ cells. The increased frequency of dark central granulation abnormalities, considered to occur during the early phases of oocyte maturation, observed in patients with low AMH levels also supports this conclusion. Cytoplasmic inclusions that do not affect oocyte development were observed more frequently in the group with normal AMH levels (17).

The authors of the present study also evaluated the morphological characteristics of the mature oocytes obtained during treatment. They evaluated the relationship between AMH levels and oocyte quality, which are considered to affect pregnancy conception. Except for the wide perivitelline space abnormality, oocyte morphology was not different between the groups with high or low AMH levels. In the meta-analysis performed by Setti *et al* on the relationship between abnormal oocyte morphologies and ICSI results, the wide perivitelline space was found to significantly decrease an oocyte’s likelihood of being fertilized (18). This finding is in line with those reported by Figueria (19). An observed wide perivitelline space has been associated with over maturity.

In conclusion, basal AMH levels can be used as a guide to predict ovarian response during IVF treatment cycles. AMH is useful in predicting the number of oocytes, the number of mature oocytes, and the number of oocytes that can be fertilized during treatment, but it is not a predictor of oocyte quality or pregnancy conception. This might be because in addition to oocyte quality, pregnancy conception is also affected by factors such as embryo quality, transfer technique, and endometrial receptivity (20). Furthermore, treatment success is also affected by sperm properties in patients who receive the treatment due to the male factor.
